# Renewed focus on reducing the burden of pre-eclampsia 

**DOI:** 10.2471/BLT.26.296138

**Published:** 2026-05-01

**Authors:** Jeremy Farrar, Ayman Abdelmohsen, Helga Fogstad, Janet Ginnard, Angel Mwiche, C Danilo Nápoles Méndez, Qurrat ul Ain, Petr Velebil, Anna af Ugglas, Shyam Desai, Paul Guerby, Frank Louwen, Angela Makris, Amilia Afzan Mohd Jamil, Graeme Smith, Sindhu K Srinivas, Peter von Dadelszen

**Affiliations:** a Health Promotion, Disease Prevention and Care, World Health Organization, Avenue Appia 20, 1211 Geneva 27, Switzerland.; b Sexual and Reproductive Health and Rights Branch, United Nations Population Fund, New York, United States of America (USA).; c Health Programme, United Nations Children’s Fund, Nairobi, Kenya.; d Unitaid, Geneva, Switzerland.; e Reproductive Health, Ministry of Health, Lusaka, Zambia.; f National Group of Gynaecology and Obstetrics, Ministry of Public Health, Havana, Cuba.; g Federal Government PolyClinic, Ministry of National Health Services, Regulation and Coordination, Islamabad, Pakistan.; h Perinatal Centre, The Institute for the Care of Mother and Child, Prague, Czechia.; i International Confederation of Midwives, The Hague, Kingdom of the Netherlands.; j South Asia Federation of Obstetrics and Gynaecology, Delhi, India.; k French College of Gynaecologists and Obstetricians, Toulouse, France.; l International Federation of Gynaecology and Obstetrics, Frankfurt, Germany.; m Society of Obstetric Medicine of Australia and New Zealand, Sydney, Australia.; n Malaysian Society of Hypertension and Clinical Practice Guideline Hypertension Committee, Kuala Lumpur, Malaysia.; o Society of Obstetricians and Gynaecologists of Canada, Kingston, Canada.; p Society for Maternal-Fetal Medicine, Washington DC, USA.; q Global Health Committee, International Society for the Study of Hypertension in Pregnancy, London, England.

Hypertensive disorders are the second leading direct cause of maternal death globally.^1^ Pre-eclampsia is a significant driver of this burden and a major contributor to perinatal mortality, maternal morbidity and long-term disability,^2^ with disproportionate impact in low- and middle-income countries.^3^ Pre-eclampsia affects an estimated 3–13% of pregnancies worldwide.^4^^–^^6^ Variations across regions are possibly explained by risk differences in some specific populations, inconsistent diagnostic criteria or underreporting.^7^

Our understanding of pre-eclampsia has changed considerably in the past two decades. Despite evidence that pre-eclampsia is a systemic and multiorgan disorder,^8^^,^^9^ clinical definitions have not kept pace. A recent review of 15 guidelines from 11 national and international professional organizations shows differences in definitions and diagnostic criteria and limited clarity on the supporting evidence.^10^ These differences highlight the need to develop a standardized, evidence-based definition and diagnostic criteria to ensure consistent clinical and public health actions.

To prevent eclampsia or the severe features associated with pre-eclampsia for both women and newborns, timely and accurate diagnosis leading to appropriate management is necessary.^11^ However, various factors hinder timely diagnosis, including limited awareness in the community of the importance of antenatal care, limited awareness of health workers around routine management of elevated blood pressure during pregnancy, insufficient or inadequately trained staff, and lack of medical supplies.^12^ An important starting point for improved identification is a definition based on diagnostic criteria that reflects the updated science, especially the multiorgan nature of the disease.

## Definitions of pre-eclampsia

In July 2025, the World Health Organization (WHO), in collaboration with Unitaid, convened a global technical consultation to discuss: (i) different definitions of pre-eclampsia and pathways to diagnosis in clinical practice guidelines; (ii) acceptability and feasibility of diagnostic methods, alongside their diagnostic and prognostic accuracy; (iii) country perspectives on its burden and current challenges; and (iv) women’s experiences of health services.

The consultation highlighted variation in definitions in clinical practice guidelines with inconsistent recognition of organ dysfunction beyond proteinuria, and the importance of aligning definitions with contemporary science and clinical realities, particularly in resource-strained settings.

Participants proposed changes that should be considered in any updated global definition of pre-eclampsia (Box 1).

Box 1 **Core elements that should be considered in an updated definition of pre-eclampsia**

Pre-eclampsia is a multiorgan disorder usually detected between 20 weeks gestation and six weeks postpartum, characterized by hypertension and evidence of organ dysfunction.

Evidence of organ dysfunction refers to the presence of proteinuria and/or other abnormal clinical or laboratory findings that suggest dysfunction of organs, including the placenta.

Remark: Operational details such as diagnostic thresholds, time of onset and severity will need to be developed to support implementation.

Experts emphasized that hypertension should be the primary trigger for action. Blood pressure must be measured at every clinical contact during pregnancy using an appropriate and accurate blood pressure measuring device, with clear pathways for diagnosis and management of pre-eclampsia activated when hypertension is first detected, reinforcing the importance of routine monitoring and early intervention.

Historically, proteinuria was considered central to diagnose pre-eclampsia. The experts highlighted that in some settings, the tests used to detect proteinuria remain readily available. However, current definitions often highlight proteinuria but fail to recognize that organ dysfunction in pre-eclampsia may extend beyond the kidneys.^10^ By expanding the definition to include clinical or laboratory evidence of dysfunction in other organs, including the placenta, more women can be identified and appropriately managed. Observational studies conducted in high-income countries show conflicting results. Some authors suggest that while using a broader definition of pre-eclampsia results in identifying additional cases, these women had less severe maternal and newborn outcomes.^13^ However, others state that using the broader definition was more efficient in capturing severe cases.^14^

The experts agreed that the explicit inclusion of the postpartum period in an updated definition also offers an opportunity to strengthen postnatal care policies and the quality of care including programmes and data.

## A shared commitment

In this article, we affirm our collective commitment to advancing progress in the prevention, detection and treatment of pre-eclampsia. Strengthening the global and national response requires a clearer and more consistent understanding of the condition. A review and synthesis of available and emerging evidence are needed to inform updates to diagnostic thresholds and key parameters for disease characterization, including timing of onset and severity.

On 5–8 May 2026, in Kigali, Rwanda, the United Nations Development Programme/United Nations Population Fund /United Nations Children’s Fund/WHO/World Bank Special Programme of Research, Development and Research Training in Human Reproduction will convene a global summit on pre-eclampsia.^15^ An important strategic pillar of the roadmap to emerge from this summit is the norms and standards priorities for pre-eclampsia, which given the current status of the disease definition, would prioritize the development of a standardized, evidence-based definition to support appropriate pre-eclampsia treatment across clinical settings and enhance programme implementation efforts. Next steps will involve establishing the methods to define clinical thresholds for key elements of the definition, particularly organ dysfunction, followed by identification of evidence and research gaps, and assessment of feasibility and potential impact across settings. Concurrently, a coordinated process within WHO will be undertaken to update the 11th revision of the *International statistical classification of diseases and related health problems* definition.

Implementation approaches will necessarily differ across contexts. Many health systems continue to face challenges such as limited access to diagnostics, fragmented referral pathways and underresourced antenatal and postnatal care. Norms and accompanying operational guidance will need to reflect these realities and emphasize feasibility, affordability and equity.

Pre-eclampsia and eclampsia continue to affect the lives of too many women and newborns. As new evidence and technologies emerge, an important opportunity exists to strengthen the continuity and quality of care, from early identification of hypertension and organ dysfunction to timely management and follow-up. Sustained collaboration among national governments, professional societies, academic institutions and global partners will be essential to ensure that advances in knowledge translate into meaningful improvements for women and their newborns. By working together to enhance clinical practice, reinforce integrated antenatal and postnatal care, improve data systems and support approaches that promote equity and feasibility across diverse settings, we reaffirm our shared commitment to reducing the burden of hypertensive disorders of pregnancy, particularly pre-eclampsia and eclampsia, and improving outcomes for all.
